# Radiographic features of Wu et al. type A2 congenital thumb duplication and implications for management: new subtypes and surgical strategies

**DOI:** 10.3389/fped.2025.1536872

**Published:** 2025-03-10

**Authors:** JianPing Wu, ShiJie Liao, YuQuan Li, FuLong Xu, Hai Zhao, ChenYang Li, YanHan Liu, XinWang Zhi, HongHong Lin, ZheHui Tu, LiLi Shu, JingChun Li, YiQiang Li, Federico Canavese, HongWen Xu, YuanZhong Liu

**Affiliations:** ^1^ Department of Pediatric Orthopaedics, GuangZhou Women and Children’s Medical Center, Guangdong Provincial Clinical Research Center for Child Health, GuangZhou Medical University, GuangZhou, China; ^2^ Department of Orthopedics, The First Affiliated Hospital of Guangxi Medical University, Nanning, China; ^3^ Department of Orthopedics, The Second Affiliated Hospital of Guangxi Medical University, Nanning, China; ^4^ Department of Pediatric Orthopedic, ChenZhou No.1 People’s Hospital, ChenZhou, China; ^5^ Department of Orthopedic and Traumatology, IRCCS Istituto Giannina Gaslini, Genoa, Italy; ^6^ DISC-Dipartimento di Scienze Chirurgiche e Diagnostiche Integrate, University of Genova, Genova, Italy

**Keywords:** congenital thumb duplication, Wu et al. classification, anatomy, interphalangeal and metacarpophalangeal joint alignment, surgery

## Abstract

**Objective:**

This study aimed to assess the radiographic features of patients diagnosed with congenital thumb duplication (CTD) type A2 based on the Wu et al. classification, describe the different subtypes of duplications and propose a classification system that permits identifying various surgical strategies.

**Methods:**

We evaluated 665 patients (680 thumbs) diagnosed with type A2 CTDs by examining the alignment of the interphalangeal (IP) and metacarpophalangeal (MP) joints of the primary thumb on posteroanterior (PA) radiographs. The classification system has four types: Type I (no deviation); Type II (ulnar deviation); Type III (hypertrophic epiphysis); and Type IV (convergent). Types I-IV were compared to Hung et al.'s system Type A-D (Hypoplastic, Ulnar Deviation, Divergent, and Convergent).

**Results:**

Of the 680 fingers, 436 (64.1%) were determined to be Wassel type IV while 244 (35.9%) were classified as Wassel type VII. All of the 436 fingers could be categorized according to the subtypes of the Hung et al. system; in particular, 369 (84.6%) were identified as type B, 52 (11.9%) as type D, and 15 cases (3.4%) as type C. The proposed classification system worked effectively for all CTDs (*n* = 680). 494 cases were classified as type II (72.6%), while 75 cases were classified as type I (11.0%). The remaining 111 cases were further classified as either type IV (9.3%) or type III (7.1%). The Wu et al. systems showed excellent intra-rater (0.881) and inter-rater (0.873) reliability compared to the Hung et al. systems (0.842 and 0.823, respectively).

**Conclusions:**

The proposed radiographic pathoanatomical system has the potential to improve communication and guide optimal procedure selection for different subtypes of CTD depending on the attachment of the extra digit to the main thumb and the alignment of the interphalangeal and metacarpophalangeal joints of the primary thumb (Wu et al. type A2).

**Level of evidence:**

III

## Introduction

Congenital thumb duplication (CTD) is a common hand anomaly observed in children (1–3), characterized by bony irregularities and abnormal localization of flexor or extensor tendons and thenar muscle (4–6). Therefore, surgical treatment should not only include resection of the supernumerary thumb, but also adequate bony and soft tissue reconstruction (5–9).

Wassel-Flatt type IV CTD, which is characterized by duplication at the metacarpophalangeal (MP) joint, accounts for between 35.3% and 44.9% of all CTDs (1–3, 6–8). Currently, limited studies are available on type IV alone, particularly in terms of its subtypes and surgical strategies, and most of these studies involve small sample sizes (4, 10–12). Dautel et al. operated on 17 thumbs of 16 children with type IV CTD using a patterned flap; their research demonstrated the technique was possible and effective in enhancing the size and shape of the reconstructed thumb (12). In an additional study, Hung et al. classified 43 cases of type IV CTD into four subtypes that have significant implications for surgical intervention (4). Previous studies did not examine the unique instances of type VII CTD. In these cases, an extra thumb appears as a triphalangeal digit, but not as the primary one. The surgical techniques for type VII CTD are similar to those for type IV. It is essential to recognize that the additional digits may attach to the main digits through a joint, cartilage, epiphysis or soft tissue, which might impact the option for surgical intervention. To date, no study has assessed the radiographic properties of CTD at the MP joint level.

To solve some of these issues, Wu et al. introduced a radiographic system capable of classifying all CTDs with excellent interobserver and intraobserver reliability (13). In particular, type A2 in Wu et al. classification includes all types of type IV CTD, which feature duplications connected by joint rather than cartilage, epiphysis, or soft tissues at the MP joint level described by Hung et al. (4). Additionally, Wu et al. classification can include certain type VII CTDs in which the supernumerary thumb is triphalangeal, but not the main thumb, and is connected to the main finger by an articulation (1–3, 8, 13).

The purpose of this research was to describe the radiographic features of patients with CTD type A2 grouped by Wu et al. classification, recognize various subtypes of type A2 CTD, and suggest a categorization approach to distinguish diverse surgical strategies based on the radiographic anatomy of this common subtype of duplication.

## Materials and methods

After obtaining approval from the Institutional Review Board (IRB) at our institution (316B01) and informed consent from parents/legal guardians of study participants, we conducted a retrospective review of medical records for 665 children (*n* = 680 thumbs) diagnosed with type A2 CTD according to the Wu et al. classification at our institution between August 2015 and April 2021 (13).

The study enrolled patients who met the following inclusion criteria: (1) a confirmed diagnosis of type A2 CTD according to the classification system developed by Wu et al.; (2) complete clinical and radiological data; and (3) treatment solely performed at our institution. Patients with incomplete clinical and radiologic data, CTD other than type A2 according to Wu et al. classification, and those treated elsewhere were excluded.

### Radiological assessment

The Wassel-Flatt classification system divides CTD into seven subtypes based on the anatomic level of skeletal duplication (3): I (bifid distal phalanx), II (duplicated distal phalanx), III (bifid proximal phalanx), IV (duplicated proximal phalanx; most common subtype), V (bifid metacarpal), VI (duplicated metacarpal), and VII (triphalangeal thumb).

**Figure 1:**
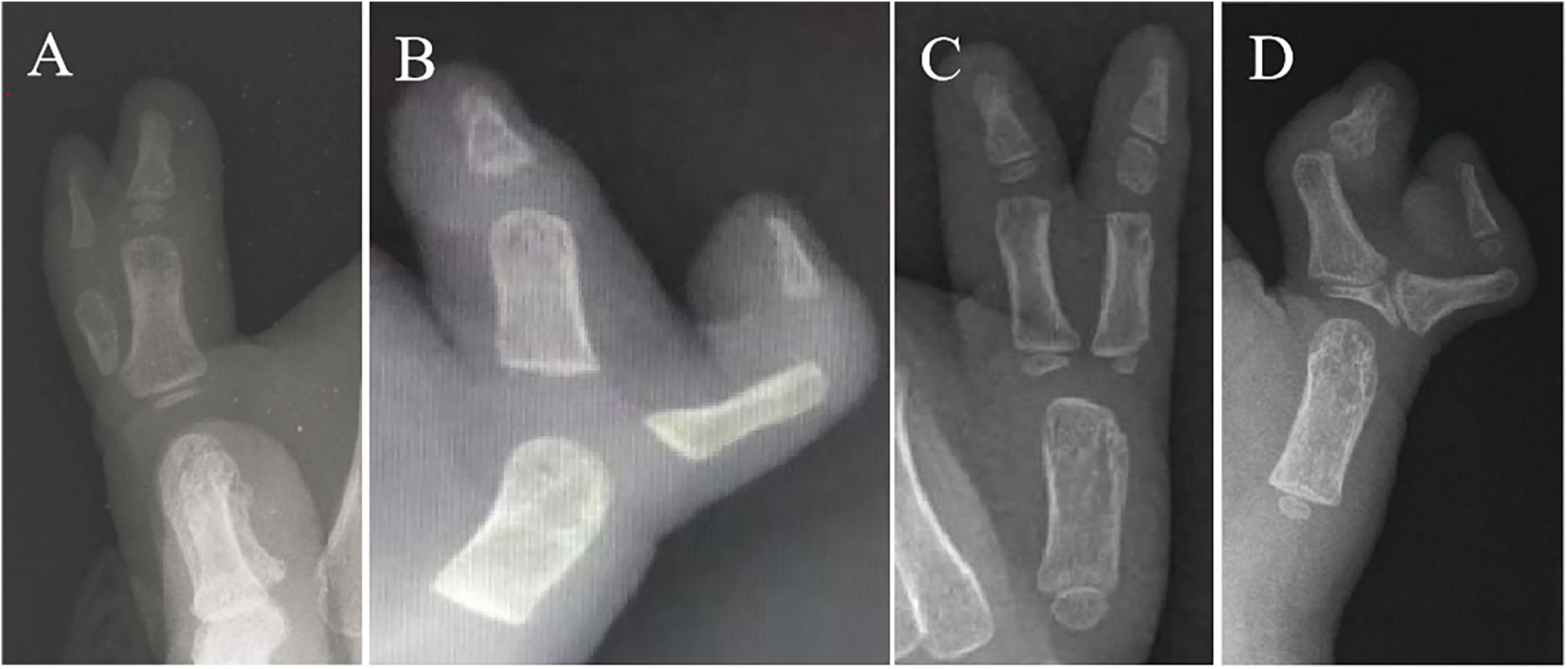
Classification system of Wassel type IV subtypes by Hung et al: type A (hypoplastic), type B (ulnar deviation), type C (divergent), and type D (convergent) (3, 4).

The classification system of Hung et al. divides CTD at the level of the MP joint into 4 subtypes (4): Type A (hypoplastic) identifies cases in which the duplicated radial thumb connected to the ulnar thumb by soft tissue is hypoplastic, atrophic, or even dangling. Type B (ulnar deviated) includes cases with a slightly hypoplastic and angulated radial thumb joined to a large ulnar thumb with or without ulnar deviation of the MP joint and interphalangeal (IP) joint. Type C (divergent) identifies cases in which both thumbs are of equal size and are straight and divergent. Type D (convergent) identifies cases in which the two thumbs joined by joint, epiphysis, or cartilage are widely separated and divergent at the MP joint, while at the interphalangeal (IP) joint the distal phalanges are acutely divergent and point toward each other, like a lobster's claws (Figure 1).

**Figure 2:**
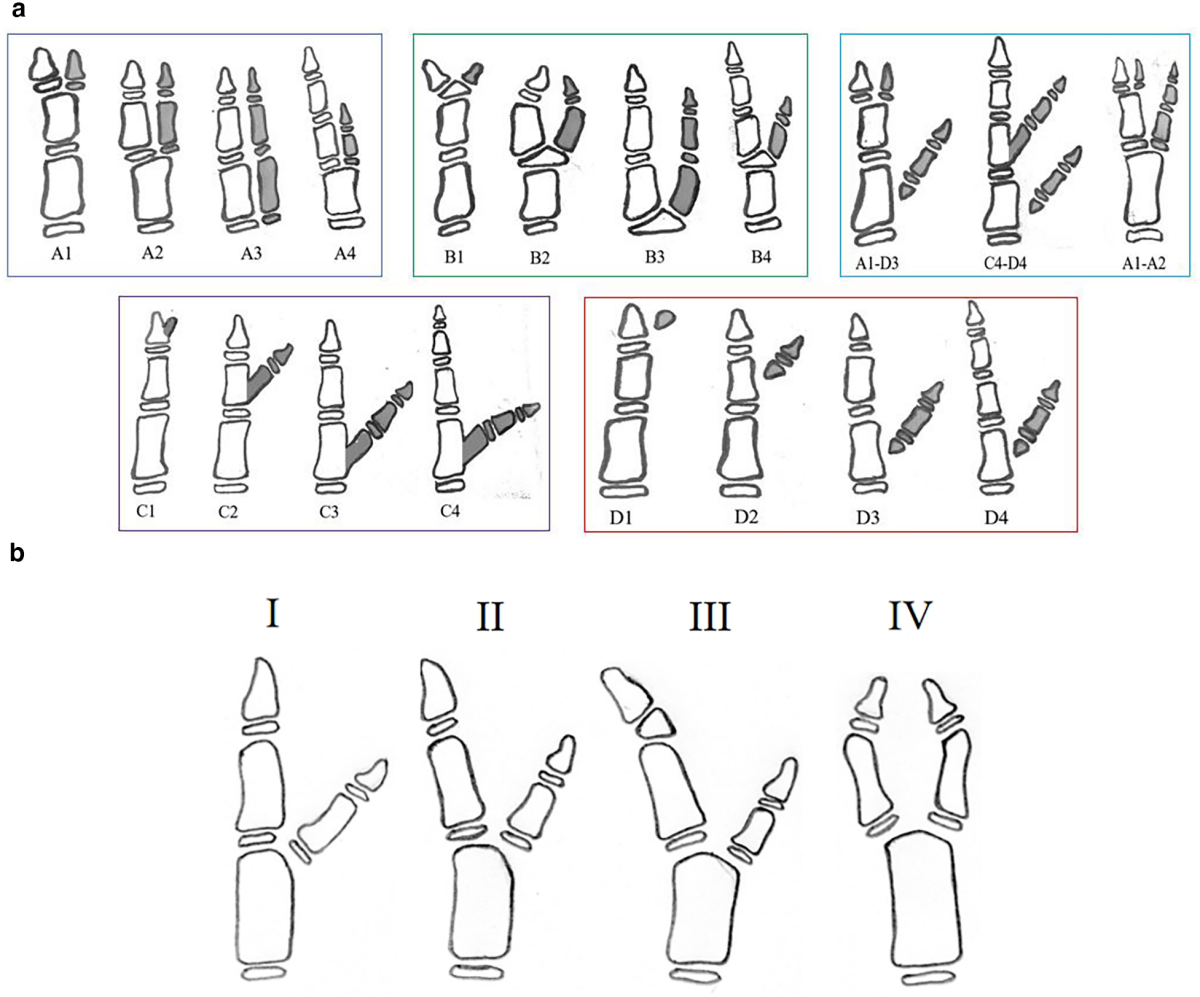
**(a)** Wu et al. classification system (13); **(b)** Congenital thumb duplication (CTD) type A2 based on the Wu et al. classification: Type I (no deviation), Type II (ulnar deviation), Type III (hypertrophic epiphysis) and Type IV (convergent).

According to the pathoanatomy at the origin of the extra thumb, the classification of CTD by Wu et al. is characterized by four main groups: A (articular type), B (epiphyseal type), C (bone type), and D (soft tissue type). Each group has four subtypes according to the location of the duplication, with the first three subtypes extending from the distal phalanges to the metacarpals or from the interphalanges to the metacarpals, and the fourth subtype including only the triphalanges of the main thumbs, whether or not the extra thumbs are present. When two or more types of extra thumbs attached to the main thumb are present, they are classified by subtypes of each extra thumb from the distal phalanx to the metacarpal (combined type) (13) (Figure 2a).

### Wu et al. A2 CTD subclassification system

The authors propose a new system for classifying the different subtypes of Wu et al. A2 CTD, in which the extra thumb is connected to the main thumb by a joint at the level of the MP joint (13). The identification of the different subtypes is essentially based on the alignment of the IP and MP joints of the main thumb, but not of the supernumerary thumb. The classification considers that angulation less than 10° as acceptable or no deviation and identifies 4 subtypes:

**Type I** (no deviation) identifies cases in which there is no axial deviation at the level of the IP and MP joints; the alignment of the IP and MP joints of the main thumb is good, with no radial and ulnar deviations;
**Type II** (ulnar deviation) identifies cases in which there is ulnar deviation of the main thumb at the level of the MP joint, while the IP joint is well aligned;
**Type III** (hypertrophic epiphysis) identifies cases where the main thumb's IP and MP joints deviate towards the ulna in association, with hypertrophic epiphysis of the distal phalanx;
**Type IV** (convergent) identifies cases with radial deviation of the IP joint and ulnar deviation of the MP joint of the main thumb (Figure 2b).

### Surgical strategies

The classification system of type A2 CTD subtypes is essentially based on the alignment of the IP and MP joints. Understanding the radiographic and clinical anatomy of the duplication can assist in selecting the optimal surgical approach.

**Figure 3:**
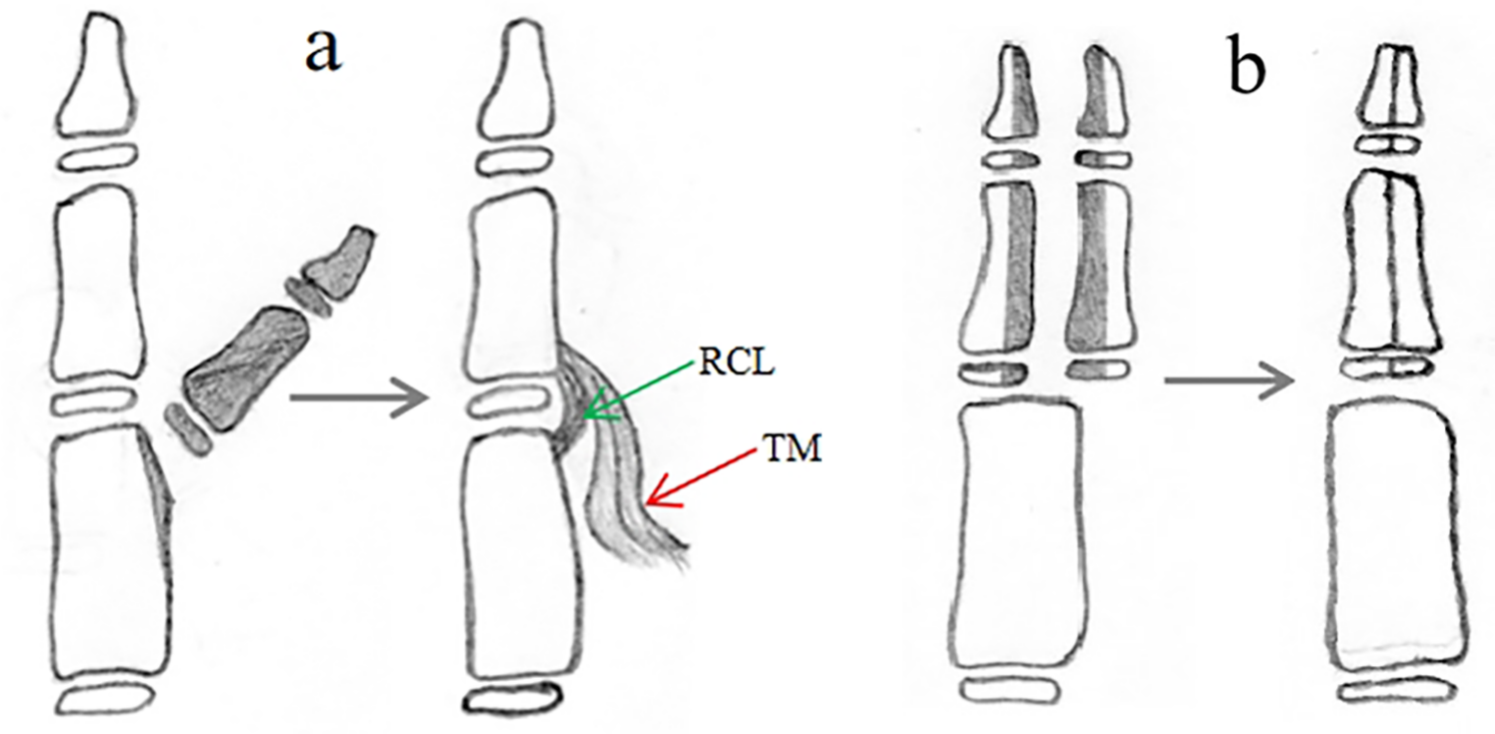
Surgical reconstruction of a type I duplication can be achieved with two procedures: **(a)** resection of the extra thumb and reconstruction of the collateral ligament and thenar muscle; **(b)** Bilhaut-Cloquet procedure, with or without nail bed reconstruction.

For type I duplications with good alignment of the IP and MP joints of the main thumb, the surgical option is mainly excision of the supernumerary thumb and reconstruction of the collateral ligament and thenar muscle of the main thumb (Figure 3a) (6, 7, 12). Some CTDs with hypoplastic duplications where both thumbs are of comparable size should be treated with the Bilhaut-Cloquet procedure (Figure 3b) (14–17).

**Figure 4:**
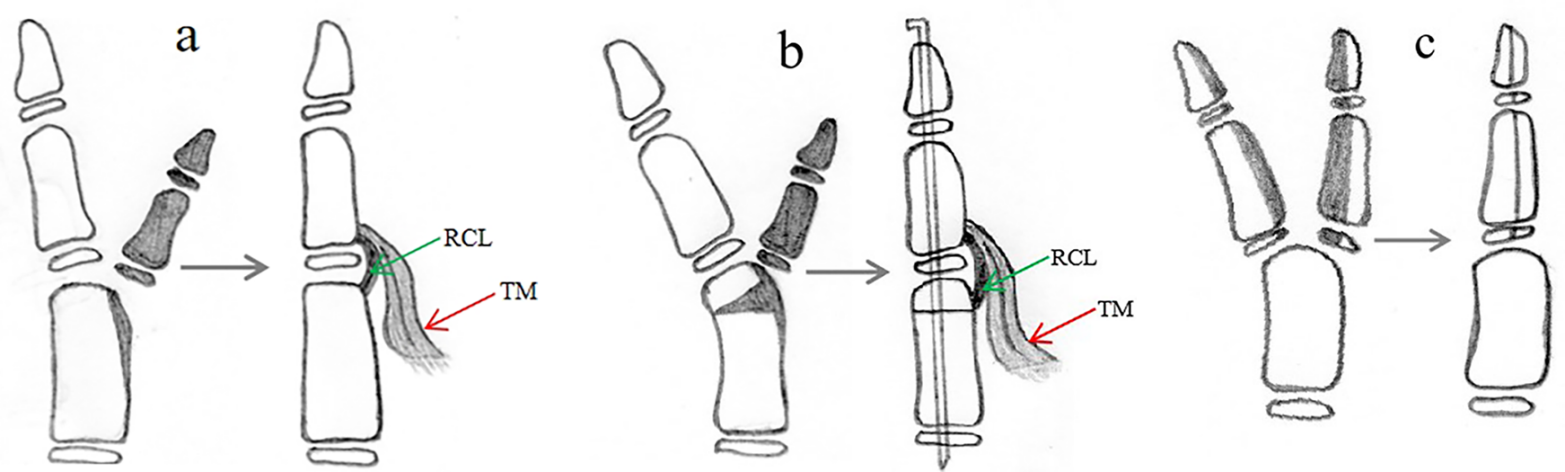
Surgical reconstruction of a type II duplication can be achieved with three procedures: **(a)** removing the extra thumb and reconstructing the collateral ligament and thenar muscle; **(b)** excising the extra thumb, reconstructing the thenar muscle, performing a corrective osteotomy of the metacarpus, and reconstructing the ligament/periosteal sleeve; **(c)** Bilhaut-Cloquet procedure, with or without nail bed reconstruction.

For type II duplications with no deviation of the IP joint and ulnar deviation of the MP joint of the main thumb, excision of the supernumerary thumb, reconstruction of the collateral ligament of the MP joint of the main thumb, osteotomy of the metacarpus and reconstruction of the periosteal ligament/sleeve may be performed as the primary option (Figure 4a,b) (17–19). Another option in this group, similar to type I forms, is the Bilhaut-Cloquet procedure for patients with hypoplastic duplications of CTDs of comparable size (Figure 4c) (14–17).

**Figure 5:**
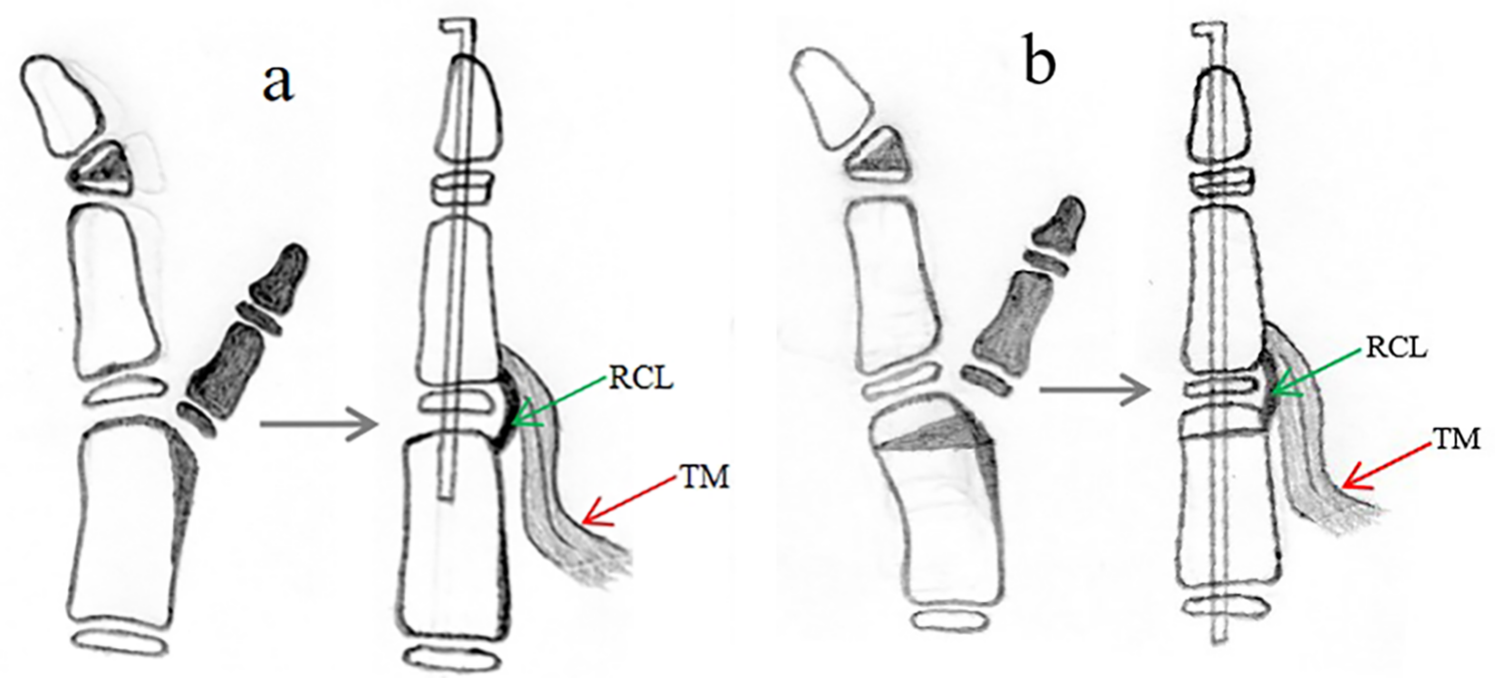
Surgical reconstruction of a type III duplication can be achieved with two procedures: **(a)** involves excision of the extra thumb, reconstruction of the collateral ligament/periosteal sleeve and thenar muscle, and corrective osteotomy of the hypertrophic epiphysis of the distal phalanx; **(b)** involves excision of the extra thumb, reconstruction of the thenar muscle, corrective osteotomy of the hypertrophic epiphysis of the distal phalanx and metacarpus, and reconstruction of the ligament/periosteal sleeve.

Type III dupliations with ulnar deviation of the IP and MP joint of the main thumb require excision of the supernumerary thumb, epiphyseal osteotomy of the distal phalanx, collateral ligament reconstruction, and reconstruction of the thenar muscle of the MP joint of the main thumb (Figure 5a); metacarpal osteotomy and periosteal ligament/sleeve reconstruction may also be performed (Figure 5b) (17–19).

**Figure 6:**
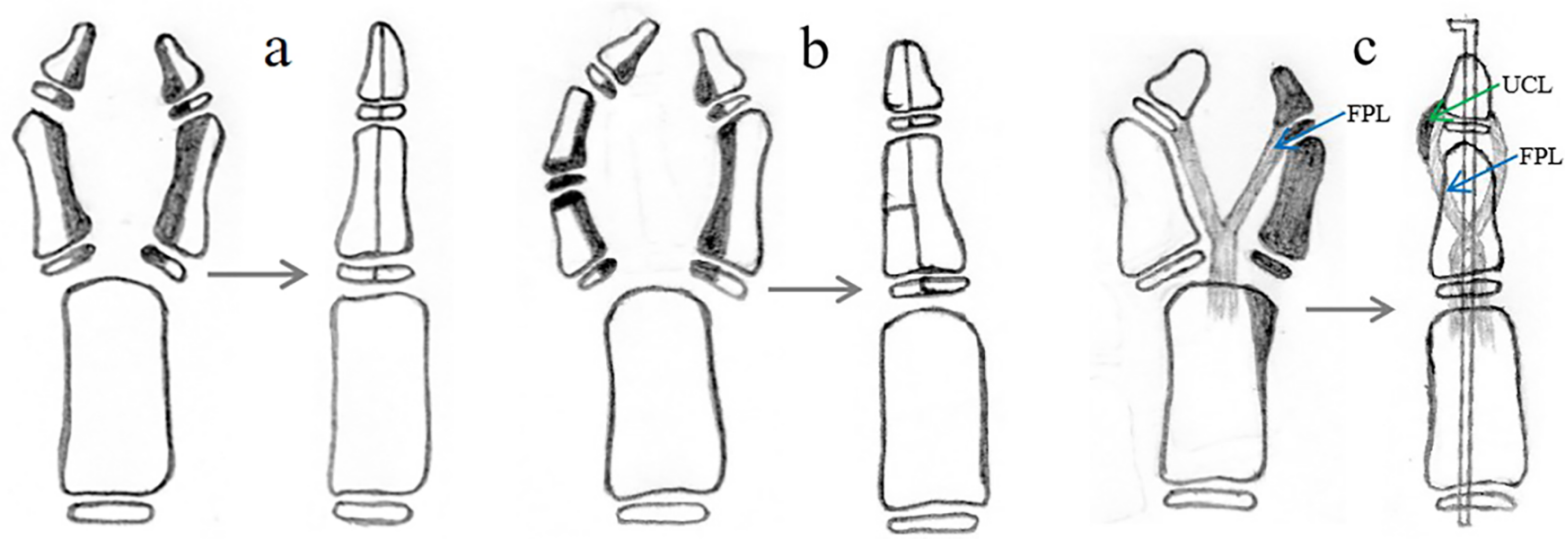
Reconstruction of type IV duplication involves the Bilhaut-Cloquet procedure **(a,b)**. Moreover, removing the extra thumb and reconstructing the ulnar collateral ligament of IP, radial collateral ligament of MP, thenar muscle, and flexor tendon reconstruction **(c)** are necessary.

Type IV duplications, which tend to present as hypoplastic duplications with the main and supernumerary thumbs of similar size and with radial deviation of the IP joint and ulnar deviation of the MP joint of the main thumb, can be successfully treated with the Bilhaut-Cloquet procedure as the primary option (Figure 6a,b) (14–17). Other options include excision of the extra thumb, reconstruction of the collateral ligament, reconstruction of the thenar muscle or flexor or extensor tendon, osteotomy of the proximal phalanx or metacarpus, and reconstruction of the periosteal ligament/sleeve (Figure 6c) (17, 20).

### Statistical analysis

All statistical analyses were performed with the SPSS 22.0 statistical package (SPSS, Chicago, IL, USA). Categorical parameters are expressed as frequencies and percentages. Quantitative data are expressed as mean ± standard deviation and range. Chi-squared test was used to compare sex, side and size of thumb duplications in these groups. The tests were two-tailed, and a *p*-value of <0.05 was considered significant.

The kappa coefficient for correlated data and the proportion accuracy (%) were calculated to measure the interobserver reliability at the first and second grading and the intraobserver reliability between the first and second grading. According to the ICC values described by Koo and Li, the agreement was interpreted as follows <0.5 (poor agreement), 0.5–0.75 (moderate agreement), 0.75–0.9 (good agreement), and 0.9–1.0 (excellent agreement) (21).

## Results

A total of 250 female and 415 male patients with CTD (*n* = 680 thumbs) met the inclusion criteria; 421 (62%) and 259 (38%) patients had the right and left sides affected, respectively. The male to female ratio and right to left ratio were 1.7:1 and 1.6:1, respectively. The average age at time of surgery was 22.9 ± 13.6 months (range, 7–140).

Among the thumbs included in the study, 436 and 244 out of 680 were classified as type IV and type VII, respectively, according to the Wassel-Flatt system while all 680 thumbs were classified as type A2 in accordance with the Wu et al. Classification.

Out of the 436 fingers that fell into the type IV of the Wassel-Flatt classification system, 64 (14.7%) had duplication of the thumb without any deviation of the IP and MP joint of the main thumb. All 436 cases were classified as Hung et al. type B (*n* = 369; 84.7%), type C (*n* = 15; 3.4%), and type D (*n* = 52; 11.9%).

All 680 cases of CTDs were classified using the Wu et al. system; specifically, 75 cases were classified as type I (11%), 494 cases as type II (72.6%), 48 cases as type III (7.1%), and 63 cases as type IV (9.3%).

Table 1 detail the patient demographics, including sex, laterality, size of comparable duplications, and type of surgical procedures according to the Hung et al. and Wu et al. system (Table 1).

**Table 1:** Hung et al. and Wu et al. system according to demographic characteristics of patients.

Characteristics n, %		Sex		Laterality		Hypoplastic duplications of comparable size		Surgical procedures	Total
		Male	Female	Right	Left	No	Yes		
Hung et al. system	A	-	-	-	-	-	-	-	-
	B	217 (58.8)	152 (41.2)	218 (59.1)	151 (40.9)	351 (95.1)	18 (4.9)	ER: 353 CO: 16	369 (70.0)
	C	8 (53.3)	7 (46.7)	10 (66.7)	5 (33.3)	9 (60.0)	6 (40.0)	ER: 15	15 (3.4)
	D	37 (71.2)	15 (28.8)	29 (55.8)	23 (44.2)	19 (36.5)	33 (63.5)	ER: 50 CO: 2	52 (11.9)
Total		262 (60.1)	174 (39.9)	257 (58.9)	159 (41.1)	379 (86.9)	57 (13.1)	-	436
chi-squared		3.193		0.589		106.457		-	-
*p* value		0.203		0.745		<0.001		-	-
Wu et al. system	I	40 (53.3)	35 (46.7)	47 (62.7)	28 (37.3)	64 (85.3)	11 (14.7)	ER: 75	75 (11.0)
	II	313 (63.4)	181 (36.6)	301 (60.9)	193 (39.1)	473 (95.7)	21 (4.3)	ER: 465 CO: 29	494 (72.6)
	III	28 (58.3)	20 (41.7)	36 (75.0)	12 (25.0)	48 (100)	0 (0)	ER: 44 CO: 4	48 (7.1)
	IV	45 (71.4)	18 (28.6)	37 (58.7)	26 (41.3)	20 (31.7)	43 (68.3)	ER: 61 CO: 2	63 (9.3)
Total		426 (62.6)	254 (37.4)	421 (61.9)	259 (38.1)	605 (89.0)	75 (11.0)	-	680
chi-squared		5.345		3.977		240.328		-	-
*p* value		0.148		0.264		<0.001		-	-

ER, Simple excision and soft tissue reconstruction; CO, Corrective osteotomy; BC, Bilhaut-Cloquet procedure.

Table 2 compare the proportions of the alignment characteristics of the IP joint and MP joint of the primary thumb in patients with CTD as per the classification systems of Hung et al. and Wu et al. (Table 2).

**Table 2:** Alignment of the interphalangeal and metacarpophalangeal joints of the main thumb following the Hung et al. and Wu et al. systems.

IP alignment Characteristics (Wu et al. classification)		Deviation level				Total
		No	IP	MP	Both	
Hung et al. system	A	-	-	-	-	-
	B	64 (17.3)	-	287 (77.8)	18 (4.9)	369 (84.7)
	C	-	-	15 (100)	-	15 (3.4)
	D	-	-	-	52 (100)	52 (11.9)
Total		64 (14.7)	-	302 (69.3)	70 (16.0)	436
chi-squared		232.903				-
*p* value		<0.001				-
Wu et al. system	I	75 (100)	-	-	-	75 (11.0)
	II	-	-	494 (100)	-	494 (72.6)
	III	-	-	-	48 (100)	48 (7.1)
	IV	-	-	-	63 (100)	63 (9.3)
Total		75 (11.0)	-	494 (72.7)	111 (16.3)	680
chi-squared		1,360.000				-
*p* value		<0.001				-

IP, interphalangeal; MP, metacarpophalangeal.

The Wu et al. systems showed excellent intra-rater (0.881) and inter-rater (0.873) reliability compared to the Hung et al. systems (0.842 and 0.823, respectively).

## Discussion

The current paper presents a new system for classifying the different subtypes of Wu et al. A2 CTD, in which the extra thumb is connected to the main thumb by a joint at the level of the MP joint (13). The classification system is based on the alignment of the IP and MP joints of the main thumb, but not of the extra thumb, and identifies 4 subtypes: no deviation (type I), ulnar deviation (type II), hypertrophic epiphysis (type III), convergent (type IV). The vast majority of cases were type II (72.6%), followed by type I (11%), type IV (9.3), and type III (7.1%). Communication between surgeons dealing with this deformity may be improved by the reported classification system.

Hung et al. classified Wassel type IV duplication, the most common type of thumb duplication, into 4 types: (A) hypoplastic, (B) ulnar deviated (the most common), (C) divergent, and (D) convergent (the most complex bone and soft tissue anomalies). This classification system has important implications for surgical management. However, the subtype system introduced by Hung et al. is not comprehensive with respect to the pathoanatomy at the origin of the extra digit and the alignment of the IP and MP joints of the main thumb. Therefore, its use in surgical practice is somewhat limited (4). Our research found that the A2 CTDs of Wu et al. can be classified into four subtypes (I, II, III, and IV) according to the alignment of the IP and MP joints of the primary thumb. In addition, it can provide some guidelines to guide their surgical treatment (13).

The modified Wassel classification proposed by Tada et al. (6), which excludes the triphalangeal thumb from the Wassel classification and replaces the floating thumb with type VII, appears to be simpler and more accessible than the modification proposed by our system. Tonkin et al. also suggested that it may be preferable to describe the duplication by skeletal level (types I-VI), noting the presence of radial and/or ulnar thumb triphalangism when applicable (22). Although the modifications of the Wassel system by Tada et al. and Tonkin et al. are relatively simple and practical, they do not take into account the anatomical characteristics of the duplication, as the extra thumb may be attached to the main thumb by bone, joint, epiphysis, cartilage or soft tissue, which has implications for the choice of surgical treatment.

Our study showed that Wu et al.'s type A2 CTD is highly prevalent in boys with right-sided tendency, which is consistent with most previous reports. In particular, Hung et al. (*n* = 43), Patel et al. (*n* = 41), Chew et al. (*n* = 10), Hong et al. (*n* = 78), and Luangjarmekorn et al. (*n* = 45) reported a male-to-female ratio between 1.4–9.0 and 1 (4, 10, 11, 18, 20), whereas in our series (*n* = 680), the ratio was between 1. 7 and 1; in addition, these previous studies reported a right-to-left ratio between 1.0–1.4 and 1, while in our series the ratio was between 1.6 and 1. These discrepancies may be related to differences in ethnicity, sample size, environmental factors, level of medical care, environment and diet, and economic status (4, 11–13, 18, 19).

Of the 680 fingers included in our study, 436 (64.1%) were type IV and 244 (35.9%) were type VII according to the Wassel et al. system, while according to the Hung et al. system, type B CTD was the most common, followed by type D, type C, and type A. However, Hung et al. type B CTDs include Wu et al. type I, II and type III CTDs, which have different anatomical specificities and different patterns of deviation and therefore require different surgical management, and in our opinion represent distinct entities (3, 4, 13). The pathoanatomical characteristics of Hung et al. type D, in which the phalanges may be connected to the main finger by joint, cartilage, or epiphysis, and type A, in which the phalanges may be connected to the main finger by soft tissue, may also influence the choice of surgical treatment. For type A duplications, the surgical option is just excision of the supernumerary thumb and reconstruction of the thenar muscle without reconstruction of the collateral ligament and metacarpal osteotomy and periosteal ligament/sleeve reconstruction (4). For type D duplications, the surgical option is excision of the extra thumb, reconstruction of the collateral ligament, reconstruction of the thenar muscle or flexor or extensor tendon, osteotomy of the proximal phalanx or metacarpus, and reconstruction of the periosteal ligament/sleeve (16, 17, 20). And the Hung et al. system is based on both the development and deviation of the duplications, but not on the morphologic characteristics of the main thumb and can mislead surgeons and make it difficult to determine whether the surgical strategies should be excision of the supernumerary thumb and soft tissue alone or should include phalanx and metacarpal osteotomy (4). In addition, Horii et al. classified the CTD bifurcation at the MP joint level into 4 types based on the details of the bifurcation shape and the connection of the radial thumb to the ulnar components by either cartilage, joint, or fibrous tissue: (A) broad cartilaginous connection between the phalanges, (B) two separate phalanges, (C) cartilaginous connection to the metacarpal, and (D) fibrous connection to the joint capsule (23). However, the classification has relative utility in clinical decision making because, for example, Horii et al. type B CTDs include Wu et al. type I to IV CTDs, which have different anatomical characteristics and may require different surgical procedures.

In this study, we proposed a classification system based on radiographic analysis of the alignment of the IP and MP joints of the first thumb and their anatomy. All CTDs could be classified according to this system; in particular, the most frequent subtype was the one with only MP joint (type II; 72.6%), followed by the one with of both the IP and MP joints (type I; 11%), the one with radial deviation of the IP joint and ulnar deviation of the MP joint (type IV; 9.3%), and the one with ulnar deviation of both the IP and MP joints with hypertrophic epiphysis of the distal phalanx (type III; 7.1%). This finding is important because the alignment of the IP and MP joints of the main thumb and the duplication characteristics may influence the choice of surgical treatment. In addition, the size of the main thumb may also influence the choice of treatment. Dobyns et al. and Townsend et al. suggested that a main thumb nail width of at least 80% of the normal contralateral thumb is acceptable (24, 25). They also noted that a thumb girth that was one-third less or greater than the normal thumb resulted in a poor cosmetic outcome. Tonkin et al. and Baek et al. suggested a Bilhaut-Cloquet procedure when the preserved thumb is less than 70% and 67% of normal width, respectively (26, 27). We agree that the Bilhaut-Cloquet procedure should be performed to obtain a normal-sized thumb in patients with hypoplastic duplications of comparable size and less than 70% of the normal side. However, the procedure has limitations, such as the technical difficulty of joining all segments of a duplicated thumb, possible subsequent physeal growth arrest, joint stiffness, and nail plate deformity (15–17). In our series, we found that type IV, type I, and type II duplications in thumbs of comparable size could be treated with the Bilhaut-Cloquet procedure in 68.3%, 14.7%, and 4.3% of cases, respectively.

In cases of type I deformity, treatment usually involves excision of the accessory thumb and reconstruction of the collateral ligament and thenar muscle of the main thumb in 85.3% of patients (6, 7, 12). For certain CTDs with similarly sized hypoplastic duplications less than 70% of the normal side, the Bilhaut-Cloquet procedure can be performed. This involves reconstructing both the distal and proximal phalanges with soft tissue reconstruction and was applicable to 14.7% of patients.

For patients with type II deformity, surgical treatment involves excision of the extra thumb, reconstruction of soft tissues such as the collateral ligament and thenar muscle, and corrective osteotomy of the metacarpophalangeal joint if the soft tissue procedure alone does not result in a thumb without MP joint deviation (Figure 4b).

Luangjarmekorn et al. found that corrective osteotomy of the metacarpus is recommended for Wassel type IV thumbs with deviation of the MP joint exceeding 30° (19). However, Gao et al. propose that corrective osteotomy should be performed if the deviation exceeds 15° (17). The Hong et al. study demonstrated that when the preoperative deviation angle of the MP joint exceeds 10.8°, patients with correctable MP joint by intraoperative radial stress test may benefit from metacarpal osteotomy (18). However, the preoperative angle was not measured using a standard anteroposterior or posteroanterior thumb radiograph, and thus may not accurately reflect the true anatomical deviation. Therefore, further clinical research with larger sample sizes and longer follow-up of patients with type II CTDs should include a standard thumb radiograph before surgery to obtain a reliable indication for metacarpal osteotomy. For our patients, we recommend that soft tissue reconstruction is sufficient if the alignment of the metacarpus appears good on a standard thumb AP radiograph before surgery and if the MP joint can be corrected via an intraoperative radial stress test. In cases where the MP joint is uncorrectable by this method, metacarpal osteotomy should be performed. In addition, some patients with hypoplastic duplications of comparable size should be treated with the Bilhaut-Cloquet procedure necessitates tissue reconstruction, with or without nail bed reconstruction.

Surgical treatment for patients with type III deformity must take into account the complex pathoanatomy of the deformity, including bony deformity with ulnar deviation of the IP and MP joints, as well as abnormal location of the thenar muscle. The surgical procedure involves excision of the extra thumb, corrective osteotomy of the hypertrophic epiphysis of the distal phalanx, ligament/periosteal sleeve, and thenar muscle reconstruction, with indications for metacarpal osteotomy identical to that of type II. Surgery may be delayed until the thumb is large enough for osteotomy of the hypertrophic epiphysis (Figure 5a,b). In addition, Johnson et al. found that CTDs with a small interspace distance, close to the size of the non-duplicated thumb and with one phalanx much smaller than the other, ar more likely to share an epiphysis. On the other hand, the more divergent and distant the duplicated bones are, the more likely they are to have separate epiphyses, which helps the surgeon to more accurately predict the type of duplication, to predict the outcome, and to strengthen the preoperative plan (28).

For patients with type IV deformity characterized by complex pathoanatomy, including bony deformity resulting in ulnar deviation of both IP and MP joints, abnormal location of tendons and thenar muscle, and hypoplastic duplications of comparable size in most patients (68.3%), the Bilhaut-Cloquet procedure (Figure 6a,b) is the optimal choice (14–17). Some patients (31.7%) without hypoplastic duplications of comparable size or rejection of the Bilhaut-Cloquet procedure may benefit from excising the extra thumb and then undergoing reconstruction of the ulnar collateral ligament of the IP, radial collateral ligament of the MP, thenar muscle, and flexor tendon (Figure 6c) (20). Moreover, few patients, particularly older children, may require corrective osteotomy of the phalanx or metacarpus, as well as ligament/periosteal sleeve reconstruction, if the IP or MP joint cannot be corrected during an intraoperative ulnar or radial stress test. Most patients who are candidates for the Bilhaut-Cloquet procedure tend to have poor function of the interphalangeal joint prior to surgery, and a postoperative range of motion of 10°–30° has been reported in previous studies to be sufficient for daily activities; we believe that appearance is at least as important as function in these patients (14, 16).

The study has some limitations. Although the new classification system has some potential advantages over previously published systems, it has not yet been evaluated for surgical treatment guidance because the Wassel-Flatt classification remains the universally accepted method for categorizing the pathoanatomy of thumb polydactyly and guiding surgical procedures. In addition, the current study was designed to evaluate the radiographic anatomy of the deformity rather than the outcomes of specific surgical procedures. Furthermore, only 9% of patients had their extra thumb removed and reconstructed with a procedure other than the one we propose, such as the Bilhaut-Cloquet procedure and corrective osteotomy of the hypertrophic epiphysis of the distal phalanx or metacarpus. Although the current study is descriptive and retrospective, it includes a relatively large number of patients with Wu et al. type A2 CTD, covering all subtypes. It is also important to note that the Wu et al. classification is a radiographic system, similar to the Wassel-Flatt classification in this respect, but more comprehensive and complex. Whether the new system will prove to have practical implications for the surgical treatment of Wu et al. type A2 CTD subtypes remains to be determined.

Therefore, additional clinical studies with large sample sizes and long-term follow-up of patients treated with different procedures are needed to verify their dependability and practicality.

## Conclusion

The proposed system is based on radiographic pathoanatomy and complements the one created by Wu et al. It has the potential to improve communication among professionals. However, more research is needed to establish how to use classification to guide surgery for children with Wu et al.'s type A2 CTD. Our results are preliminary, so while the proposed system has the potential to benefit surgical strategy, further investigation is necessary.

## Data Availability

The original contributions presented in the study are included in the article/Supplementary Material, further inquiries can be directed to the corresponding authors.
